# A Full‐Range Proximity‐Tactile Sensor Based on Multimodal Perception Fusion for Minimally Invasive Surgical Robots

**DOI:** 10.1002/advs.202502353

**Published:** 2025-06-19

**Authors:** Dongsheng Li, Tianci Ji, Yuyang Sun, Zhongbin Zhang, Aomen Li, Mengjiao Qu, Dongze Lv, Jin Xie, Huicong Liu

**Affiliations:** ^1^ School of Mechanical and Electrical Engineering Jiangsu Key Laboratory of Embodied Intelligence Robot Technology Soochow University Suzhou 215137 China; ^2^ State Key Laboratory of Fluid Power and Mechatronic Systems Zhejiang University Hangzhou Zhejiang 310027 China

**Keywords:** full‐range proximity‐tactile sensor, multimodal perception, piezoelectric micromachined ultrasonic transducers (pMUTs), robot‐assisted minimally invasive surgery (RMIS), subcutaneous tissue diagnosis, vibration feedback

## Abstract

Minimally invasive surgical robots have received widespread attention due to its numerous advantages. However, the lack of adequate perception capability remains a significant issue for the robots. In this work, a full‐range proximity‐tactile sensing module has been developed for safe operation of surgical robots, which performs multimodal fusion perception through ultrasonic sensor for long‐range proximity detection, capacitive sensor for close‐range proximity sensing, and triboelectric sensor for tactile sensing. In order for a minimum sensor size, the ultrasonic sensor is developed based on MEMS piezoelectric micromachined ultrasonic transducers (pMUTs), and the capacitive sensor and triboelectric sensor adopt common structures, which collaborate to achieve accurate proximity‐tactile perception. Additionally, a wireless vibration feedback wristband and digital‐twin interface are developed to provide multimodal feedback without interfering with operation. Experimental results demonstrates the safety enhancement for surgical robots by the perception and feedback system. Furthermore, the sensing module is applied in preliminary detection of subcutaneous abnormal tissues and the identification accuracy based on the ultrasound echoes and convolutional neural networks is 91.6%, which can provide an initial diagnostic reference. The full‐range proximity‐tactile sensor holds significant potential for enhancing the safety and detection capability of surgical robots, and promoting the intelligence of robot‐assisted minimally invasive surgery.

## Graphical abstract

A full‐range proximity‐tactile sensing module is developed for safe operation of surgical robots, which performs multimodal fusion perception through ultrasonic sensor for long‐range proximity detection, capacitive sensor for close‐range proximity sensing, and triboelectric sensor for tactile sensing. Meantime, the sensing module can be applied in preliminary detection of subcutaneous abnormal tissues, providing an initial diagnostic reference.

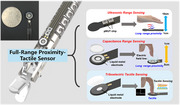

## 1 Introduction

Robot‐assisted minimally invasive surgery (RMIS) has emerged as a preferred surgical option.^[^ ^1^, ^2^, ^3^, ^4^ ^]^ Compared to traditional surgeries, minimally invasive surgery (MIS) offers many advantages, including reduced trauma, faster recovery, and fewer complications.^[^ ^5^, ^6^, ^7^, ^8^ ^]^ Surgical robots enhance the precision of surgeons’ operations and provide 3D visual feedback,^[^ ^9^, ^10^, ^11^, ^12^ ^]^ thereby reducing surgical errors and demonstrating great potential in complex MIS procedures. In recent years, robotic systems such as the da Vinci system have shown significant efficacy in improving surgical outcomes and reducing intraoperative risks.^[^ ^13^, ^14^, ^15^, ^16^ ^]^

However, most RMIS are performed in complex environments and surgeons typically rely solely on visual cues and their surgical experience. The lack of adequate perception capability remains a significant issue for the robots,^[^ ^17^, ^18^, ^19^ ^]^ which makes it difficult for the operator to accurately judge the distance between the robots and delicate tissues.^[^ ^20^ ^]^ When robotic surgical instruments are in close proximity to human tissues, the absence of tactile feedback can lead to unintentional tissue damage or abrasion,^[^ ^21^, ^22^, ^23^, ^24^ ^]^ which is particularly pronounced in narrow anatomical regions, limiting the safety and precision of surgical procedures.^[^ ^25^, ^26^, ^27^, ^28^ ^]^ Moreover, relying solely on visual information for tissue examination is insufficient for detecting subcutaneous tissue lesions, and only biopsy can be performed, causing pain to the patient.^[^ ^29^, ^30^ ^]^ Chen et al. designed a skin‐like ion‐conductive sensor adheres to the surface of a continuum robot, enabling tactile sensing capabilities for the robot.^[^ ^31^ ^]^ Li et al developed a tactile sensor array based on optical waveguide for detecting multiple contacts force in MIS.^[^ ^32^ ^]^ These two sensors have successfully achieved precise and real‐time contact detection of medical robotics. However, single‐modal sensing capability of tactile sensors cannot fully meet the safety requirements of surgical robot operations.

Surgical robots need to issue timely warnings before contact with human tissues to ensure operation safety. Therefore, proximity detection is equally critical as a key safeguard for the surgical robots. To facilitate convenient self‐localization and intelligent perception in soft robots, Shi et al. integrated ultrasonic sensors and triboelectric tactile sensor into the robotic hand.^[^ ^33^ ^]^ Nonetheless, at close distances, the ultrasonic sensor has considerable positioning and distance detection errors due to signal overlap and interference, which leads to decreased accuracy. Liu et al. developed a flexible bimodal skin integrated into soft robots, which incorporates a triboelectric sensor for touchless sensing and a liquid metal sensor for tactile sensing.^[^ ^34^ ^]^ However, the non‐contact sensing range of the triboelectric sensor is limited. Currently, achieving full‐range sensing from long‐range proximity to close‐range proximity and contact remains a significant challenge. Additionally, the end effector of surgical robot typically operates through narrow channels, which imposes stringent size constraints on sensors and introduces new challenges in their design and fabrication. Therefore, the realization of full‐range proximity and tactile sensing for surgical robots remains a critical challenge.

**Figure 1:**
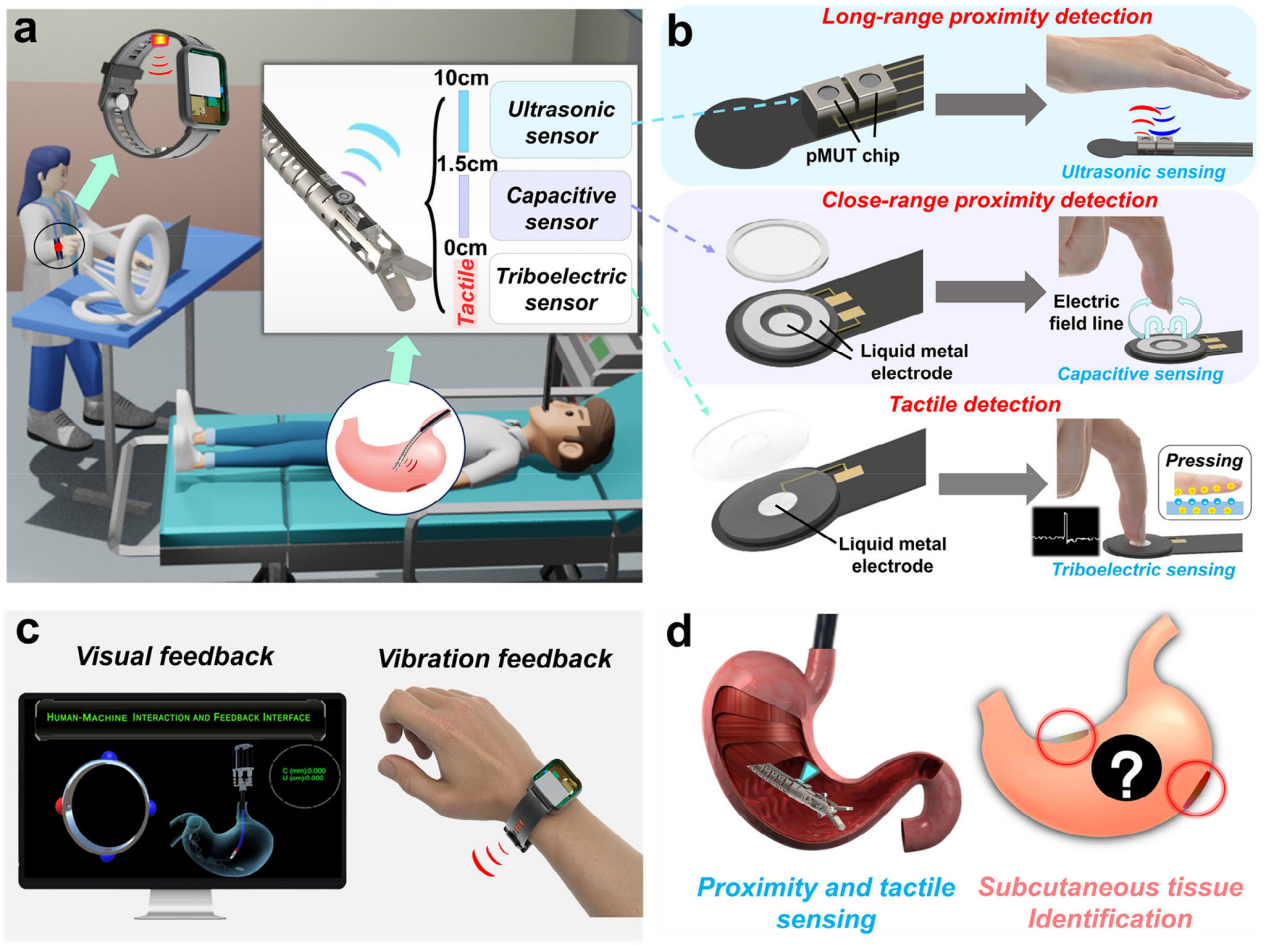
a) Schematic diagram of workspace of the full‐range proximity‐tactile sensor integrated onto MIS robot. b) Structure and working principle of the sensing module. c) Structure of the wireless force feedback wristband and the visual/vibration feedback system. d) Schematic diagram of subcutaneous tissues identification based on the proposed sensor.

To address these challenges, a full‐range proximity‐tactile sensing module was developed and integrated onto the surface of surgical robot, as shown in **Figure** **1**a. As shown in Figure 1b, the sensing module combines ultrasonic sensor, capacitive sensor, and triboelectric sensor to achieve precise multimodal perception. The ultrasonic sensor based on MEMS piezoelectric micromachined ultrasonic transducers (pMUTs) performs long‐range proximity detection and robot spatial positioning.^[^ ^35^, ^36^, ^37^ ^]^ Capacitive sensor and triboelectric tactile sensors adopt common structures and ensures accuracy in close‐range proximity sensing and provides real‐time tactile sensing capability, respectively. Based on MEMS technology and common structure design, the sensing module maintains a small size while achieving multimodal perception. Additionally, as shown in Figure 1c, we developed a wireless vibration feedback wristband and digital‐twin interface, which receives real‐time sensing signals and provides vibration feedback, alerting surgeons to adjust the robot's posture and enhancing the safety of surgical operations. Furthermore, the sensing module was applied in preliminary detection of subcutaneous abnormal tissues of the human body based on the different acoustic impedances of various tissue materials, providing surgeons with an initial diagnostic reference, as shown in Figure 1d. Based on analysis of ultrasonic signals using convolutional neural networks (CNN), the proposed sensor achieves accurate identification of different subcutaneous tissues. The full‐range proximity‐tactile sensor holds significant potential for enhancing the safety and detection capabilities of surgical robots.

## 2 Results and Discussion

### 2.1 Long‐Range Proximity Detection Based on pMUTs

**Figure 2:**
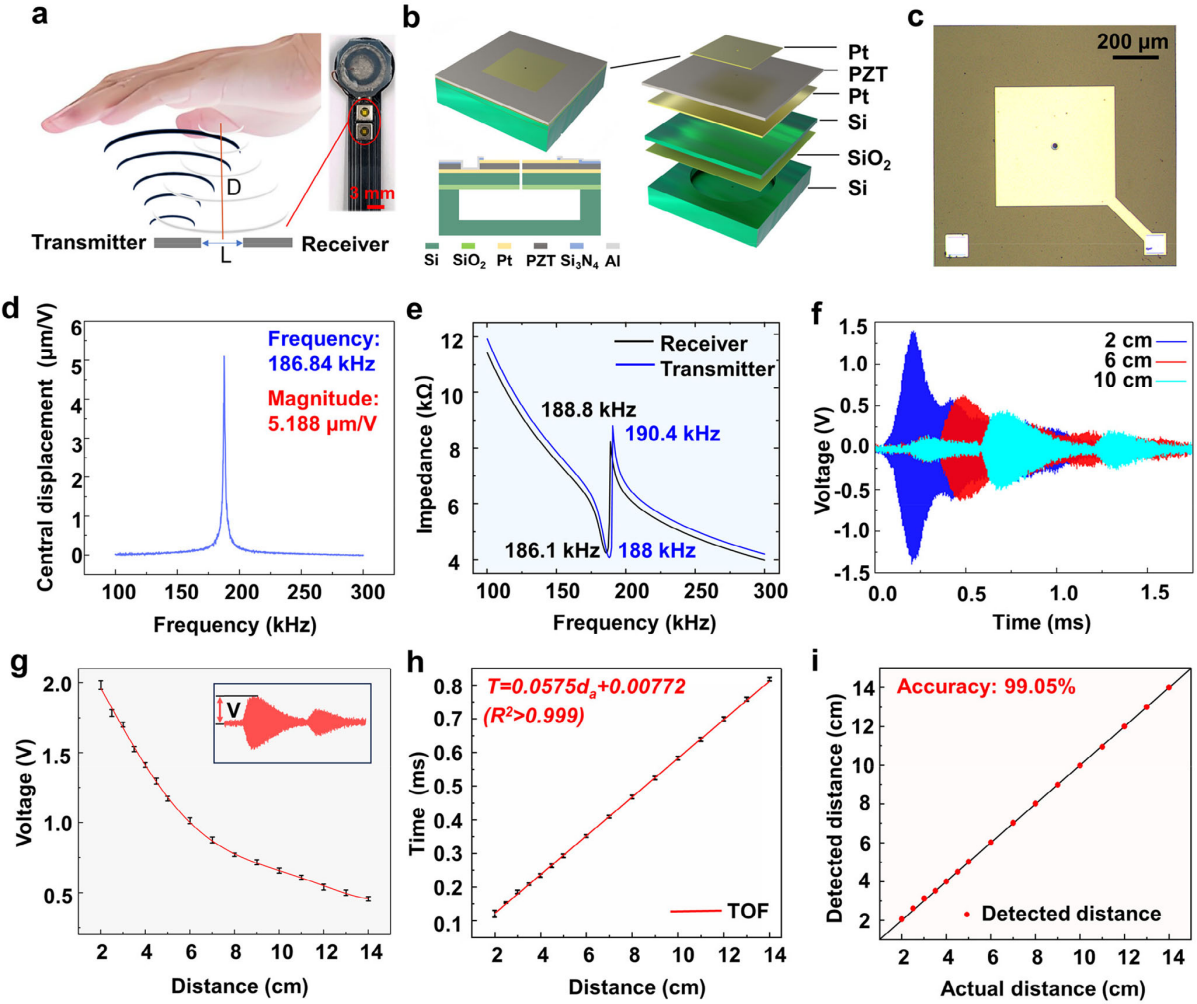
a) Schematic diagram of the distance detection principle of the pMUTs, b) Cross‐sectional and exploded diagram of the pMUT, c) Optical microscope image of the pMUT chip, d) Relationship curve between center displacement and frequency, e) Impedance response curves of the transmitter and receiver pMUTs, f) Ultrasonic echo signals at different reflection distances, g) Peak amplitude of echo signals at various distances, h) Variation of TOF with reflection distance, i) Comparison between actual distance and detected distance.

**Figure** **2**a illustrates optical image of the full‐range sensing module and the principle of long‐range proximity sensing based on pMUTs. The ultrasonic proximity sensor employs a single‐transmitter (Tx) and single‐receiver (Rx) configuration, which are responsible for emitting ultrasonic waves and capturing ultrasonic echo signals, respectively. By measuring the time difference between the transmission and reception of the ultrasonic signals, known as the time of flight (TOF), the distance between the robot and human tissue can be calculated. Since there is a certain distance (*L*) between the two pMUTs, the propagation distance *D* of the ultrasound is not merely twice the distance between the sensor and the tissue but also includes the separation between the sensors. The ultrasound propagation distance (*D*) is given by;^[^ ^38^ ^]^

(1) $$D=\frac{\sqrt{v^{2}t^{2}-L^{2}}}{2}$$

where *v* is the speed of sound, *t* is the TOF. Figure 2b shows the sectional and exploded diagram of the pMUT. The lead zirconate titanate (PZT) piezoelectric layer is sandwiched between two platinum (Pt) electrodes, enabling effective conversion between electrical and mechanical energy, thereby facilitating the generation and detection of ultrasonic waves. When an excitation signal is applied to the Tx, its PZT film vibrates due to the inverse piezoelectric effect, thus emitting ultrasonic waves. When these waves are reflected and captured by the Rx, the Rx converts the vibrations into electrical signals through the piezoelectric effect. Figure 2c presents the optical microscope image of the pMUT, measuring 1.2 × 1.2 mm^2^. The pMUTs employ leadless chip carrier (LCC) packaging, resulting in an overall size of 2.8 × 2.8 mm^2^ after packaging.

The analysis of mechanical and impedance characteristics for the pMUTs were first performed. The mechanical response amplitude of the pMUT at different frequencies is shown in Figure 2d, indicating that the maximum amplitude at the pMUT chip center point was 5.188 µm/V, occurred at a frequency of 186.84 kHz. The mechanical properties of the pMUTs can ensure good performance in both transmission and reception. Figure 2e shows the impedance of the two pMUTs, indicating that the resonance frequency of the Tx is 188 kHz, while the resonance frequency of the Rx is 186.1 kHz. The two pMUTs have a good frequency matching.

Then we tested the proximity detection capabilities of the ultrasonic sensing module. Figure 2f displays the echo signals received by the pMUT at distances of 10 cm, 6 cm, and 2 cm from a reflective barrier, with different colors indicating the varying distances. In all three cases, the receiver captured two echo signals, with the amplitude of the second echo being smaller than the first, indicating that the ultrasound can reflect multiple times. As the reflective distance increased, the delay time of the echo gradually lengthened, and the peak amplitude decreased. Peak amplitudes and flight times of the echo signals at different reflective distances were tested, as shown in Figure 2g,h, demonstrating that the flight time is directly proportional to the distance, while the peak amplitude is inversely proportional to the square of the distance. As shown in Figure 2h, There is a good linear relationship between TOF and distance with a correlation coefficient (*R^2^*) exceeding 0.999, which can be described by the following Equation (2).

(2) $$T=0.0575d_{a}+0.00772$$

where *T* represents the TOF (ms), and *d_a_* represents the actual distance (cm). The result correlates closely with the calculations from theoretical Equation (1). Figure 2i compares the actual distances with those measured by the ultrasonic sensor. The points located on the diagonal indicate that the actual distance is exactly the same as the measured distance, showing high accuracy of the sensor within the range of 2 – 14 cm, with relative error of 0.95% and measurement accuracy of 99.05%. The calculation method is illustrated in Figure S1 (Supporting Information).

Meanwhile, the sensor demonstrates excellent stability and repeatability (Figure S2, Supporting Information), with measured standard deviation (SD) of 0.0515 cm and relative standard deviation (RSD) of 0.844% over a 14‐day period, enabling it to provide accurate real‐time distance measurement during surgical procedures. However, in the 0–2 cm range, the distance detection accuracy of the sensor drops to 82.8% due to physical limitations of the ultrasonic sensor (Figure S3, Supporting Information), illustrating that the ultrasonic sensor can achieve high‐precision distance detection over long‐range proximity detection but are somewhat limited at very close distances. Considering that human tissues may approach the ultrasonic sensor from any angle, we further evaluated its measurement accuracy at different orientations. Experimental results demonstrate that the sensor maintains over 95% measurement accuracy within ±45° (Figure S4, Supporting Information), meeting the requirements for detection in RMIS.

### 2.2 Close‐Range Proximity Sensing Based on Capacitive Sensor

**Figure 3:**
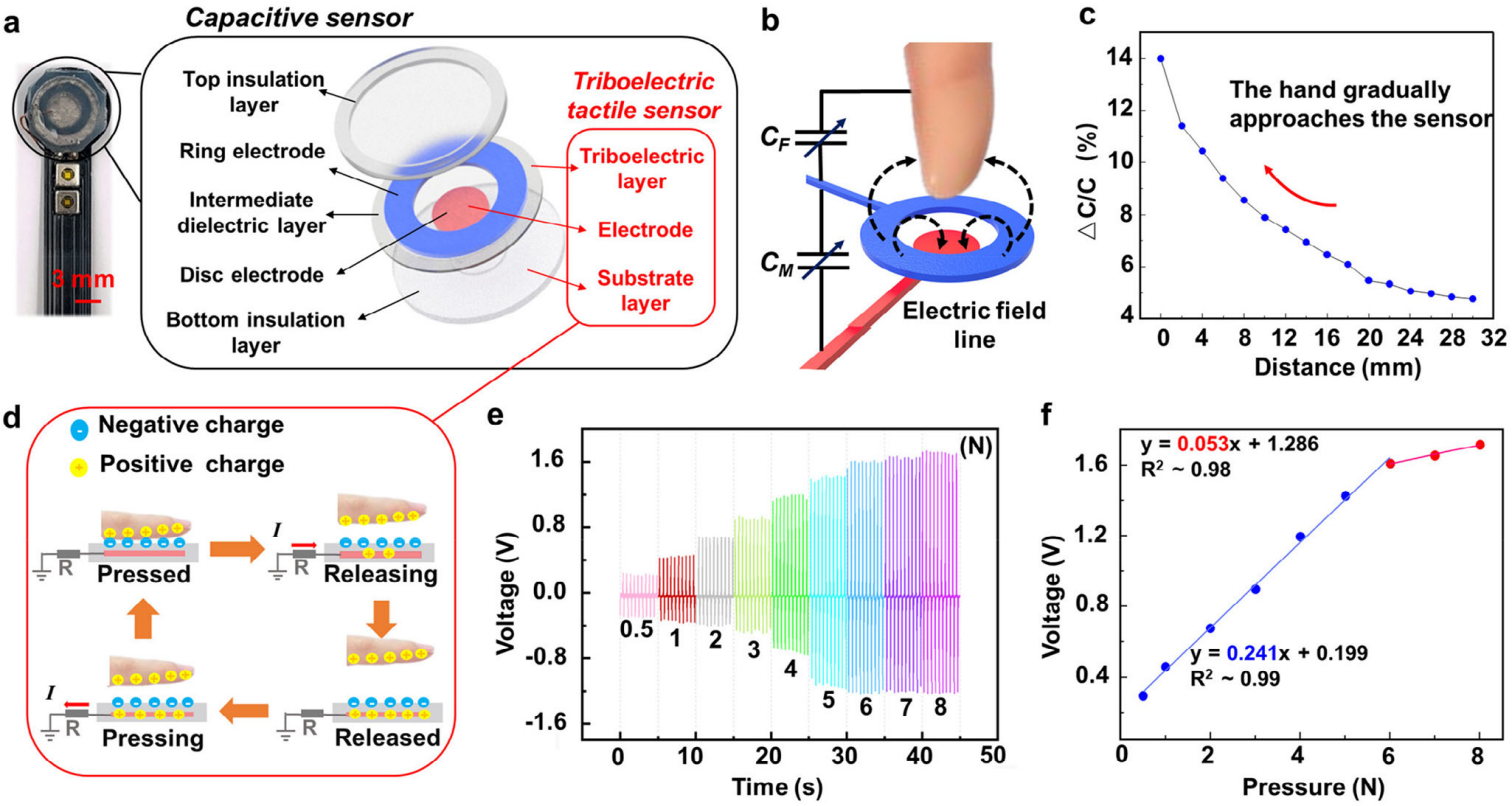
a) Detailed structure of the integrated design of the capacitive proximity sensor and triboelectric tactile sensor, b) Working principle of the capacitive sensor for close‐range proximity detection, c) Relationship curve of capacitance change with hand proximity for the capacitive sensor, d) Working principle of the triboelectric tactile sensor, e) Open‐circuit voltage of the triboelectric tactile sensor under different pressures, f) Linear relationship between applied pressure and generated voltage for the triboelectric tactile sensor, g) Open‐circuit voltage of the triboelectric tactile sensor at different frequencies.

Capacitive sensors demonstrate high sensitivity in close‐range proximity detection to fingers and other human tissues.^[39^^]^ As shown in **Figure** **3**a, a capacitive sensor was designed for close‐range sensing, consisting of two liquid metal electrodes and three layers of insulation layers of Ecoflex. The capacitive sensor was integrated and electrically connected at flexible printed circuit board (FPCB), as human tissue gradually approaches the sensor, the capacitance value changed, enabling high‐precision near‐field sensing. Figure 3b illustrates the working principle of the capacitive sensor. The ring electrode is separated from the disc electrode by the intermediate dielectric layer, creating a vertical projection field that generates a capacitance, denoted as *C_M_*. When a finger (or other human tissue) acts as a third electrode and approaches the sensor, it couples with the sensor to produce an additional capacitance, denoted as *C_F_*. As the finger approaches the sensor, its conductive nature alters the distribution of the electric field in the system. The electric field, which is originally concentrated between the electrodes, is partially diverted towards the finger, leading to a reduction in the field strength between the electrodes. This redistribution of the electric field results in a decrease in *C_M_*, while *C_F_* increases. Consequently, as the finger moves closer to the sensor, the capacitance of the sensor gradually decreases.

To characterize the performance of the capacitive sensor, we conducted experiments with a hand approaching the sensor surface from a distance. Figure 3c shows the relationship between capacitance change and the distance from the sensor surface. The sensor has proven to be highly sensitive at very close proximity. When the hand is farther from the surface, the change in capacitance decreases, but within a distance of 1.6 cm, the change is quite significant. To implement the close‐range proximity sensing of the capacitive sensor, the relationship between capacitance change and distance can be described by the following Equation (3) with a *R^2^* of 0.997.

(3) $$r=1.26/\left(d+10.2\right)+0.0157$$

where *d* and *r* represent the distance and relative variation of capacitance, respectively. Thus, the capacitance change can be accurately converted into distance. The capacitive sensor achieves real‐time close‐range proximity detection based on the Equation (3), with a measurement accuracy of 96.47% (detailed calculation process shown in Figure S5, Supporting Information).

Additional testing demonstrates that the capacitive sensor exhibits high stability and repeatability (Figure S6, Supporting Information), with measured SD of 0.119% and RSD of 1.62% over a 14‐day period, satisfying the requirements for accurate distance measurement. When an object approaches from different angles, experimental results demonstrate that the capacitive sensor maintains good accuracy (>89%) within ±15° deflection (Figure S7, Supporting Information). Furthermore, the influence of different human tissues and object shapes on sensor response has been explored, and the difference of electrical properties of different human tissues has little impact on the sensor response (Figures S8,S9, Supporting Information).

### 2.3 Tactile Detection Based on Triboelectric Sensor

In order to reduce the size of the proximity‐tactile sensor, the triboelectric tactile sensor and capacitive proximity sensor adopt common structures, with the bottom disc electrode of the capacitive sensor serving as the electrode of the triboelectric tactile sensor, as shown in Figure 1b. The triboelectric tactile sensor employs a single electrode structure, consisting of two layers of Ecoflex (triboelectric and substrate layers) and a layer of liquid metal electrode. Based on the triboelectric effect, it enables real‐time contact detection. Figure 3d shows the working principle of the triboelectric tactile sensor: When the finger/tissue touches the triboelectric sensor, due to the difference in electron affinity, electrons transfer from the surface of the finger to the surface of the Ecoflex. When the finger separates from the triboelectric sensor, the negative charges on the Ecoflex surface drive free electrons from the liquid metal electrode to the ground, resulting in the accumulation of positive charges in the liquid metal electrode. When the finger approaches the sensor again, the potential difference causes electrons to flow from the ground back to the liquid metal electrode, generating a voltage in the opposite direction. The resulting triboelectric signal manifests as a pulse signal.

The performances of the triboelectric tactile sensor were characterized under different impact forces and frequencies. As shown in Figure 3e, the open‐circuit voltage correspondingly increased from 0 V to 1.63 V as the applied pressure increased from 0 N to 8 N, which is because the applied pressure is proportional to the deformation of the top layer, resulting in an increased tactile area. The increase in charge transfer leads to greater voltage output. When the pressure increases beyond a certain level, the top layer of the sensor reaches maximum deformation, and the increase in tactile area becomes negligible, leading to saturation of the output voltage and current. Figure 3f illustrates the relationship between the applied pressure and the resulting voltage. As the pressure increases, the open‐circuit voltage rises accordingly. In the range of 0.5 N to 5 N, the linear fitting slope is 0.241 V/N, while in the range of 5 N to 8 N, the linear fitting slope is 0.053 V/N, representing the sensitivity of the sensor. To maintain high sensitivity, the sensor's applicable tactile range is defined as 0 N to 5 N, enabling accurate and rapid tactile detection. In addition, the triboelectric tactile sensor demonstrates excellent stability and repeatability, with signal amplitudes consistently maintained within the range of ‐0.296 to 0.252 V, as shown in Figure S10 (Supporting Information). The triboelectric tactile sensor is capable of providing stable signal output under repeated loading conditions.

### 2.4 Wireless Vibration Feedback Wristband

**Figure 4:**
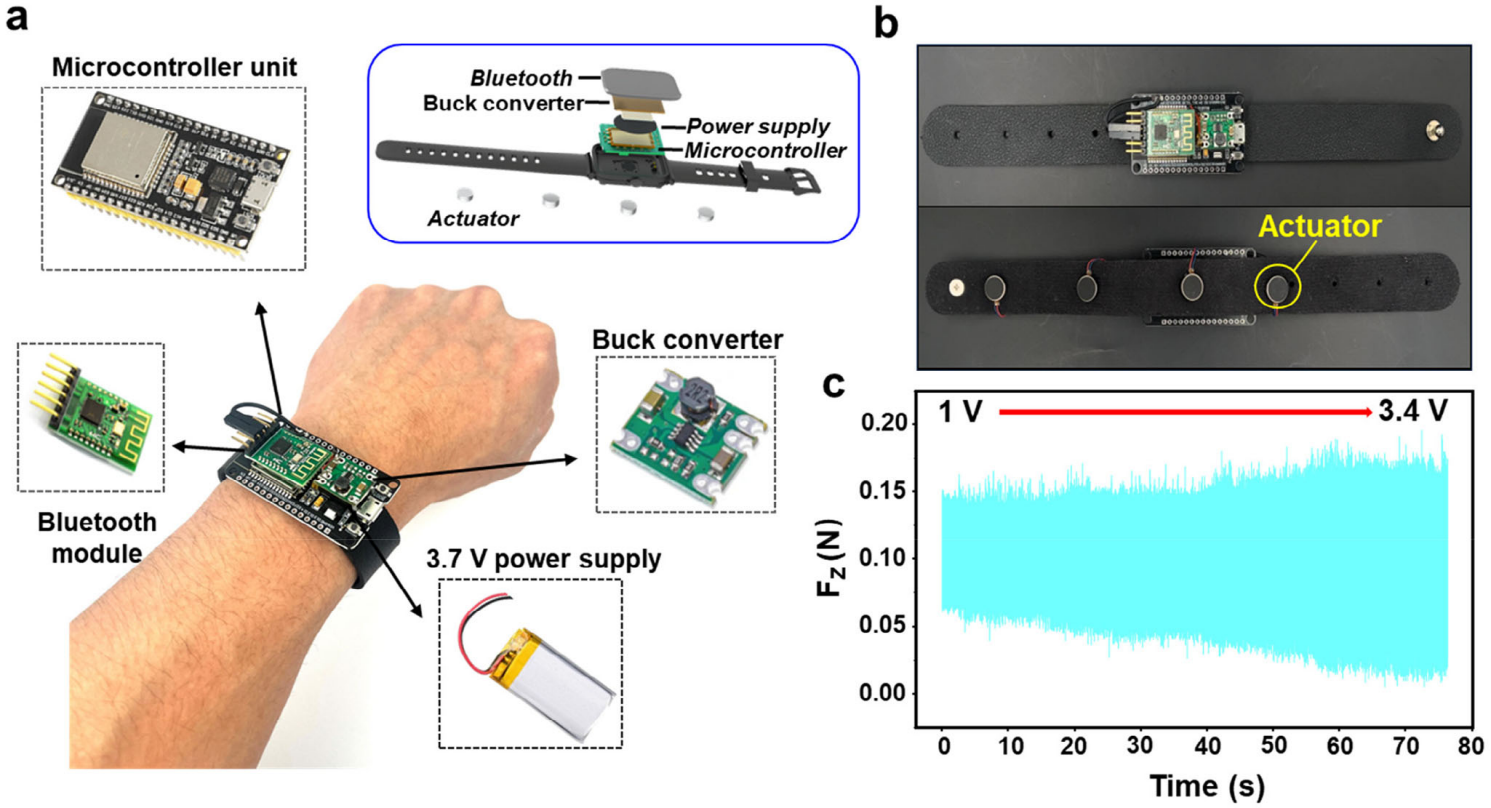
a) Detailed structure of the wireless vibration feedback wristband, b) Front and back view of the wireless vibration feedback wristband, c) Variation of vibration force with increasing applied voltage.

In order to achieve effective feedback of sensing information to operator without changing their operation logic and interfering their operations, a wireless vibration feedback wristband was designed as shown in **Figure** **4**a,b. The vibration function of the wireless feedback wristband is primarily achieved using four vibration actuators, which correspond to the four directions of up, down, left, and right respectively. When the end of the robot approaches human tissue in a certain direction, the Bluetooth transceiver module receives perception information from the proposed sensor and sends it to the microcontroller unit. Then the microcontroller unit processes the signal and send instructions to the corresponding actuator, which will emit vibration to the human body, reminding the operator. The wristband's main body also includes a DC power source for power supply.

The vibration actuators operate based on the principle of Faraday's electromagnetic induction, where an increase in voltage leads to a greater vibration amplitude, resulting in stronger vibration effects. We characterized the force output of the vibration actuators by conducting vertical force measurements with applied voltages ranging from 1 V to 3.4 V, as shown in Figure 4c. At the typical operating voltage of 3.3 V, supplied by the microcontroller unit, the actuator produced a vibration force of approximately 0.17 N, which meets the necessary intensity to provide feedback to the operator.

### 2.5 Perception and Feedback System for Surgical Robot

**Figure 5:**
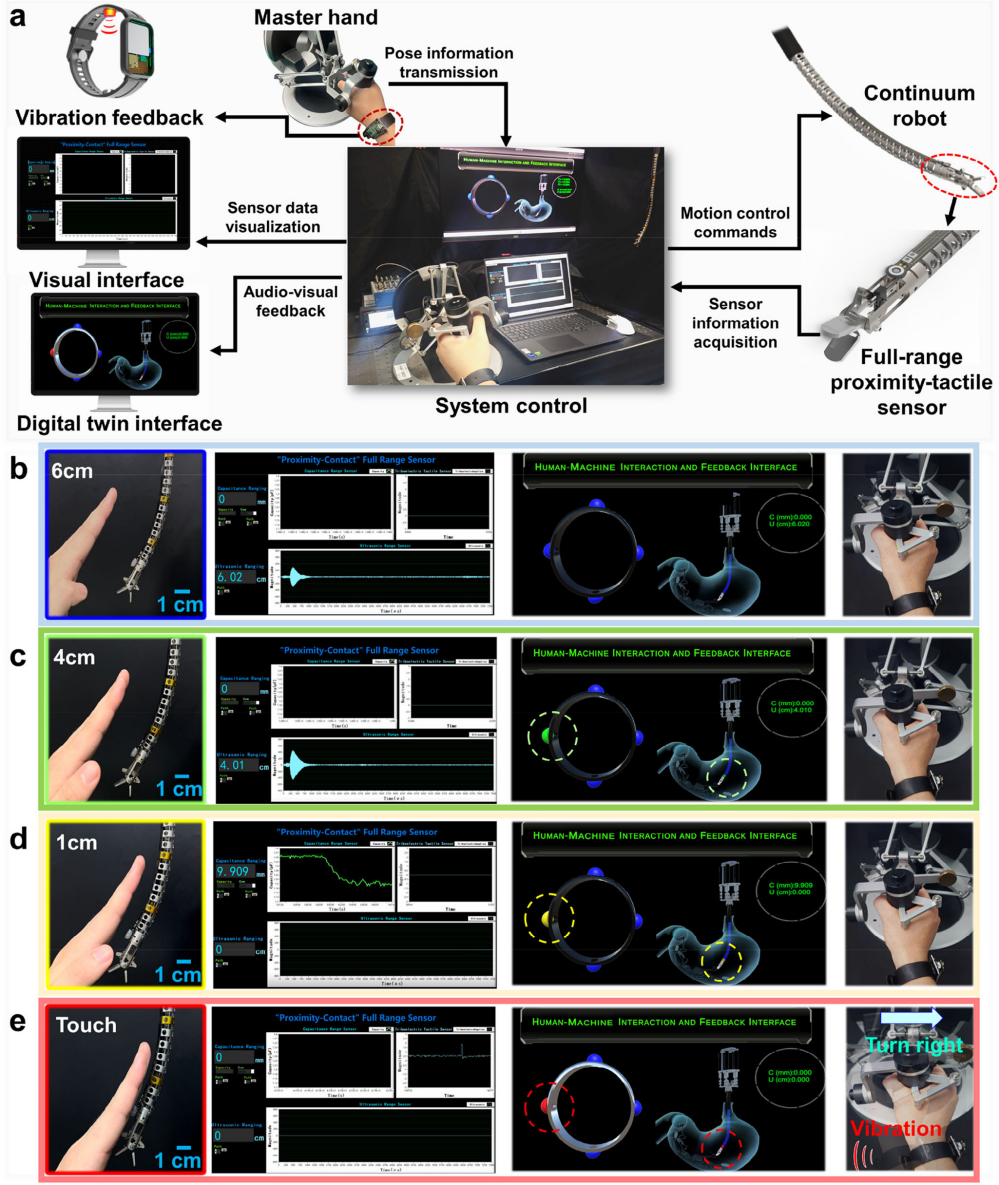
a) Diagram of the perception and feedback system, Signal visualization interface, digital twin interface, and operator when the human tissue reached distance of b) 6 cm, c) 4 cm, d) 1 cm, and e) 0 cm.

As shown in Figure S11 (Supporting Information), the ultrasonic sensor demonstrates lower measurement accuracy than the capacitive sensor for distances within 1.55 cm, whereas its accuracy surpasses that of the capacitive sensor for distances larger than 1.55 cm. Based on these characteristics, a corresponding detection strategy was developed: The pMUTs used for proximity detection within the range of 10 cm to 1.5 cm, while the capacitive sensor is employed for detection below 1.5 cm, tactile detection between robot and human tissue is realized via the triboelectric sensor. The full‐range proximity‐tactile sensor was fixed onto a flexible printed circuit board, which was then adhered to the surface of the continuum surgical robot's end effector, as illustrated in Figure S12 (Supporting Information). Consequently, a perception and feedback system was established, as shown in **Figure** **5**a. The perception‐feedback system also includes the wireless vibration feedback wristband, signal visualization interface, and digital twin interface for the robot. The ultrasonic, capacitive, and triboelectric signals are processed, and displayed in real‐time on the visualization interface. The digital twin interface not only shows the proximity‐tactile detection results but also displays the robot's bending shape and the wristband's vibration status in real‐time, providing audiovisual feedback. The wireless vibration feedback wristband receives sensing signals via Bluetooth and provides vibration feedback to the operator, enhancing the intuitiveness and responsiveness of the interaction.

The safe distance between robot and human tissue was set between 10 cm and 5 cm. Additional visual feedback was triggered within the 5 cm to 1 cm range, and the region from 0 cm to 1 cm was defined as a warning zone, accompanied by audiovisual and vibration feedback. Figure 5b–e and Video S1 demonstrated the performance of the perception and feedback system as the human tissue gradually approaches the sensor integrated onto the robot. As shown in Figure 5b, when the human tissue reached a distance of 6 cm, the ultrasonic sensor measured 6.02 cm, indicating it was still within the safe range, and operator does not need to change the posture of the continuum robot. As shown in Figure 5c, when the human tissue approached at 4 cm, the ultrasonic sensor measured 4.01 cm, and the left indicator on the wristband in the digital twin interface turned green, signifying that the system was still within the safe range, and the continuum robot's posture remained stable. When the distance was reduced to 1 cm, the ultrasonic sensor ceased functioning, and the capacitive sensor began close‐range detection, displaying a distance of 0.9909 cm. At this point, the left indicator on the wristband turned yellow, accompanied by a soft audio alert, warning the operator that the delicate tissue was near, as shown in Figure 5d. As shown in Figure 5e, upon contact with the issue, the capacitive sensor registered a distance of ≈0 mm, stopping signal acquisition, while the triboelectric tactile sensor detected and displayed the tactile signal. At this point, the left indicator on the wristband in the digital twin interface flashed red, emitting a loud warning sound, and the vibration feedback wristband activated, alerting the operator that contact had occurred. Based on the feedback, the operator promptly adjusted the master hand's posture to the right, and the continuum robot's posture was adjusted accordingly in real‐time to ensure safe operation. As the continuum robot moved away from the human tissue, the capacitive sensor and ultrasonic sensor alternated in providing real‐time distance feedback. These experimental results demonstrated that the full‐range proximity‐tactile sensing and multimodal feedback system effectively achieved proximity and tactile perception and feedback, providing reliable support for ensuring safe surgical operations.

### 2.6 Subcutaneous Tissue Identifications Based on Ultrasound Echo and CNN

**Figure 6:**
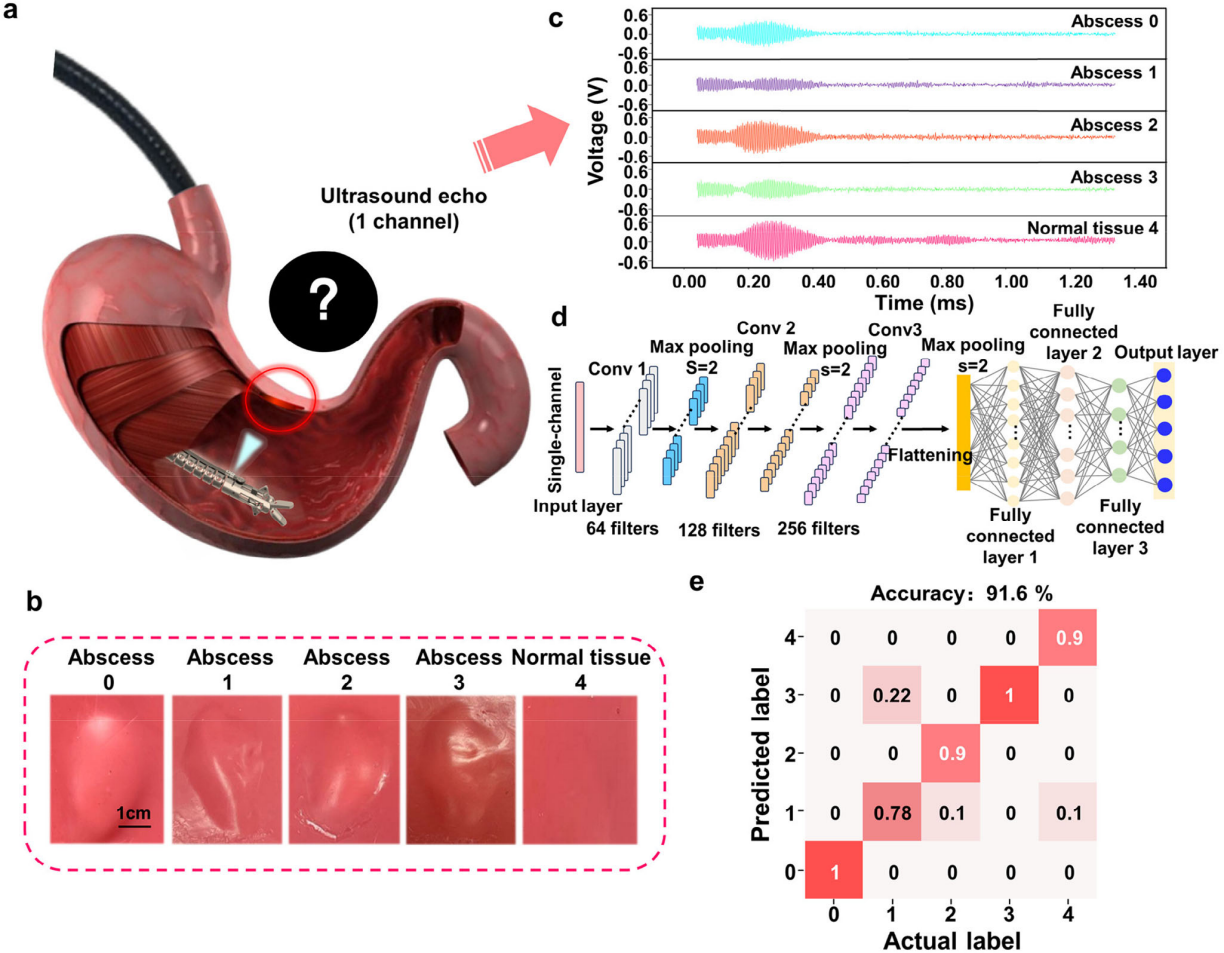
a) Schematic diagram of the identification for subcutaneous pathological tissues, b) Optical images of five different human tissue simulants, c) Ultrasonic echo signals for the five different human tissue simulants, d) Structure of the CNN model, e) Confusion matrix of the identification for subcutaneous tissues validated by LOOCV.

In surgical procedures, such as gastric surgeries, subcutaneous tissues may contain pathological entities such as tumors or abscesses, as shown in **Figure** **6**a. Tissue examination using surgical robots often relies solely on visual information, which is unable to identify subcutaneous tissue lesions. While pMUTs are capable of long‐range proximity detection, these echo patterns also show variations when interacting with various human tissues at the same distance. Therefore, the sensing module was further applied in preliminary detection of subcutaneous abnormal tissues. Ultrasound echoes from different materials exhibit significant variation due to differences in acoustic impedance, absorption properties, and structural characteristics. Acoustic impedance is determined by the material's density and sound velocity, which can affect the shape and frequency response of the echoes. Additionally, materials with higher absorption coefficients causing greater attenuation of the echo signal. Internal structures also impact the reflection and scattering of ultrasound waves.

To validate the identification performance of the pMUTs, we fabricated five types of simulated subcutaneous tissues, as illustrated in Figure 6b. The models include: Abscess 0 (containing different types of simulated muscle tissue), Abscess 1 (containing different simulated muscle tissue with DI water), Abscess 2 (containing the same type of simulated muscle tissue), Abscess 3 (containing only DI water), and Normal Tissue 4. Ultrasound echo signals were collected at the same distance from the five types of simulated tissues, with 20 samples for each type, totaling 100 samples. As shown in Figure 6c, the ultrasound echoes from normal tissue exhibited the largest peak amplitude, while Abscess 1 showed the smallest peak.

To effectively identify these tissues based on their ultrasound echo signals, a CNN model was constructed. Given that ultrasound signals are typically 1D time‐series data, we employed a 1D CNN for feature extraction, whose advantages lie in their ability to extract hierarchical features from the signal. The structure of the CNN model is illustrated in Figure 6d, the model consists of an input layer for single‐channel time‐series data, with the feature dimension corresponding to the sequence length. Three convolutional layers were included with 64, 128, and 256 filters (kernel size: 3×1), each followed by a max pooling layer (stride: 2) to reduce feature dimensions. The output was transformed into a one‐dimensional vector, which is processed by three fully connected layers with 128, 64, and 32 neurons (activation function: ReLU). The output layer, with 5 neurons and a SoftMax activation, classifies the data into five categories.

Considering the relatively small amount of data in the dataset, we adopted the leave‐one‐out cross‐validation (LOOCV) method for CNN model validation, which is well‐suited for small datasets as it maximizes the use of all available samples for model training and validation. After inputting the dataset into the CNN model for training and validation, the resulting confusion matrix is shown in Figure 6e, with an overall validation accuracy of 91.6% by LOOCV, proving good fitting and generalization abilities of the CNN models. The experimental results demonstrate that based on the proposed sensing module and CNN models and different subcutaneous tissues can effectively identified, enabling the preliminary detection of abnormal tissues, offering surgeons valuable diagnostic insights, which holds significant potential for future applications in medical diagnostics and assistance.

## 3 Conclusion

In this work, a full‐range proximity‐tactile sensing module was proposed based on perception fusion of three types of sensors. The ultrasonic sensor based on pMUTs achieved long‐range proximity detection with an accuracy of >99%. Accurate and fast close‐proximity and tactile detection were conducted by capacitive sensor and triboelectric sensor, which adopt common structures for a minimum size. Furthermore, a wireless vibration feedback wristband and digital‐twin interface were developed to form a perception and feedback system together with the proposed sensor. Experimental results demonstrated the accurate full‐range sensing ability of the sensor and the safety enhancement by the system. Additionally, the identification accuracy of subcutaneous tissues based on the sensor ultrasound echoes and CNN exceeded 91%, which can provide an initial diagnostic reference. This technology holds significant potential for enhancing the safety of RMIS, enhancing the detection capability of surgical robots, and promoting the intelligence of RMIS.

## 4 Experimental Section

### Fabrication of the Full‐Range Proximity‐Tactile Sensor

The detailed fabrication process of pMUTs was shown in Figure S13 (Supporting Information). The detailed fabrication process of capacitive and triboelectric sensor was shown in Figure S14 (Supporting Information). The triboelectric tactile sensor and the capacitive proximity sensor were integrated. The sensor fabrication process begins by applying a 200 µm layer of Ecoflex as the substrate layer. Next, a liquid metal layer was applied to the mold's circular hole (2 mm in diameter), with wires connected to the liquid metal disc. A second 200 µm layer of Ecoflex was then applied on top of the disc electrode as an intermediate dielectric layer. Subsequently, a liquid metal ring electrode was formed on the intermediate layer, with wires attached. Finally, a 200 µm layer of Ecoflex was applied as the top encapsulation layer.

### Structure and Workflow of Wireless Vibration Feedback Wristband

The main components of the wireless vibration feedback wristband were: an ESP32 microcontroller unit for processing sensor data and enabling the vibration actuator, an HC‐25 Bluetooth module used for wireless information transmission and reception, a 3.7 V to 1.5 V buck converter module (1.5V was the operating voltage for the Bluetooth module), a 3.7 V DC power supply module, and four vibration actuators with diameter of 8 mm and thickness of 2.7 mm. The workflow was implemented as follows: the wristband receives triboelectric signals transmitted by the computer's Bluetooth module. The microcontroller board, pre‐programmed with a signal threshold algorithm, enables the vibration actuators when the triboelectric tactile sensor detects contact, or the signal exceeds the predefined threshold, thereby triggering the vibration feedback.

### Experimental Characterizations and Software Platform

The input signal to the pMUT was a 186 kHz sine wave with a voltage of 10V, applied by a signal generator (KEYSIGHT 33500B). After passing through a filter module and an amplification circuit module, the ultrasonic echo was read and processed in LabVIEW via an NI data acquisition card (NI USB‐6366), and displayed on a LabVIEW visualization interface. The impedance characteristics of the ultrasonic sensor were measured using an impedance analyzer (MICROTEST 6632), with a sweep frequency range of 100 kHz to 300 kHz, and its mechanical performance was tested by applying a 1 V sine wave signal ranging from 100 kHz to 300 kHz on a laser doppler vibrometer (LDV) micro‐vibration platform (Ploy MSA‐060). The vibration actuator was fixed on a 3‐DOF linear stage integrated with a commercial 6‐axis force/torque sensor (ATI, Nano 17) and applied a voltage ranging from 1 V to 3.4 V using a DC power to investigate the variation in the force output of the vibration actuator. The capacitance sensor signal was measured and displayed processed using an LCR meter (TH2830) and the LabVIEW visualization interface. The triboelectric tactile sensor signal was read by an Arduino microcontroller unit. The data was transmitted via the HC‐25 Bluetooth transmission module, and then displayed on the LabVIEW visualization interface through the serial port. The LabVIEW visualization interface (Figure S15, Supporting Information) stores the three types of sensor signals in a document, which were transmitted in real time on the control interface of the continuum robot which was developed using C++ Visual Studio 2019. Simultaneously, the data was transmitted via transmission control protocol (TCP) communication to the Unity digital twin interface (Figure S16, Supporting Information), providing real‐time feedback on the sensor data and the wristband status. Experiments for the perception and feedback system adhere to ethical guidelines and demonstration experiments were completed by the authors without the participation of other human subjects, which meets the criteria for ethical review exemption.

### Fabrication of Continuum Surgical Robot

The structure of the continuum robot consists of two main components: the robot body and the drive system. The robot body includes two independently bendable joint segments arranged in series to form an elongated structure, along with a gripper section, providing four degrees of freedom for bending and one for the gripper opening and closing. The drive system was designed based on a combination of cable‐driven and tendon‐sheath transmission mechanisms, enabling the bending motion of the robot body and the opening and closing function of the gripper. The detailed design was shown in Figures S17 and S18 (Supporting Information).

## Conflict of Interest

The authors declare no conflict of interest.

## Supporting information



Supporting Information

Supplemental Video 1

## Data Availability

The data that support the findings of this study are available from the corresponding author upon reasonable request.
