# Supplementary material for: A Full‐Range Proximity‐Tactile Sensor Based on Multimodal Perception Fusion for Minimally Invasive Surgical Robots
Source: Adv Sci (Weinh). 2025 Jun 19;12(30):e02353. doi: 10.1002/advs.202502353 (PMC12376709; doi:10.1002/advs.202502353)
Supplement: Supplementary file 1 — Supporting Information [file ADVS-12-e02353-s001.pdf]

ADVANCED SCIENCE
Open Access

Supporting Information

for *Adv. Sci.*, DOI 10.1002/advs.202502353

A Full-Range Proximity-Tactile Sensor Based on Multimodal Perception Fusion for Minimally Invasive Surgical Robots

*Dongsheng Li, Tianci Ji, Yuyang Sun, Zhongbin Zhang, Aomen Li, Mengjiao Qu, Dongze Lv, Jin Xie and Huicong Liu**

Supporting Information

A Full-Range Proximity-Tactile Sensor Based on Multimodal Perception Fusion for Minimally Invasive Surgical Robots

Dongsheng Li[a,#], Tianci Ji[a,#], Yuyang Sun[a], Zhongbin Zhang[a], Aomen Li[a], Mengjiao Qu[b], Dongze Lv[b], Jin Xie[b], and Huicong Liu[a,*]

[a] School of Mechanical and Electrical Engineering, Jiangsu Key Laboratory of Embodied Intelligence Robot Technology, Soochow University, Suzhou 215137, China

[b] State Key Laboratory of Fluid Power and Mechatronic Systems, Zhejiang University, Hangzhou, Zhejiang 310027, China

[#] These authors contributed equally to this work.

*Corresponding author: Prof. Huicong Liu

Email: hcliu078@suda.edu.cn

Telephone Number: +86-18605128284

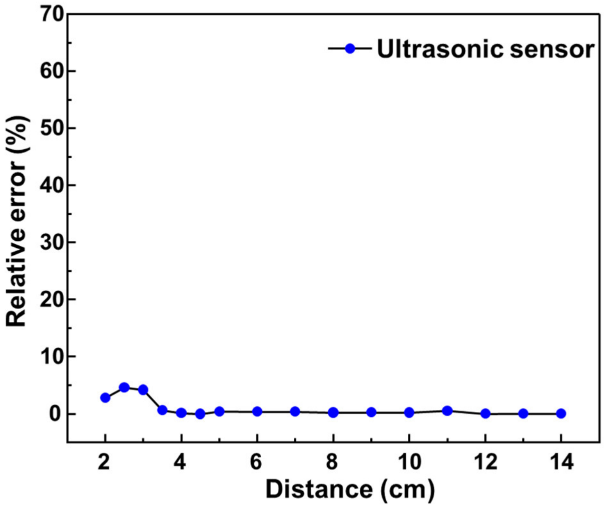

Fig. S1 Relative measurement error of the ultrasonic sensor at different distances.

The average relative error of the ultrasonic sensor is 0.95%. Ten repeated measurements were conducted at each distance, and the sensor detection results were recorded. The measured values were compared with the true values to calculate the relative errors. The average relative error of the ten measurements was then determined as the relative error at the distance. (Equation S1) The relative error of the sensor is calculated as the average of relative errors across all measured distances, from which the sensor's measurement accuracy can be derived, (Equation S2)

$$R = \frac{1}{n}\sum_{i=1}^{n}\frac{|x - x_i|}{x} \times 100\% \qquad \text{(S1)}$$

$$Accuracy = 1 - \frac{1}{k}\sum_{j=1}^{k} R_j \qquad \text{(S2)}$$

where, n is the number of detections (n=10), x is the actual value, x_i is the detection value, and R is the mean relative error of the specific distance, k is the number of detected distances, R_j is the relative error at the jth distance. Detection errors of the sensor at different distances are shown in Fig. S1. The ultrasonic sensor exhibited a relative error of 0.95%, corresponding to a measurement accuracy of 99.05%.

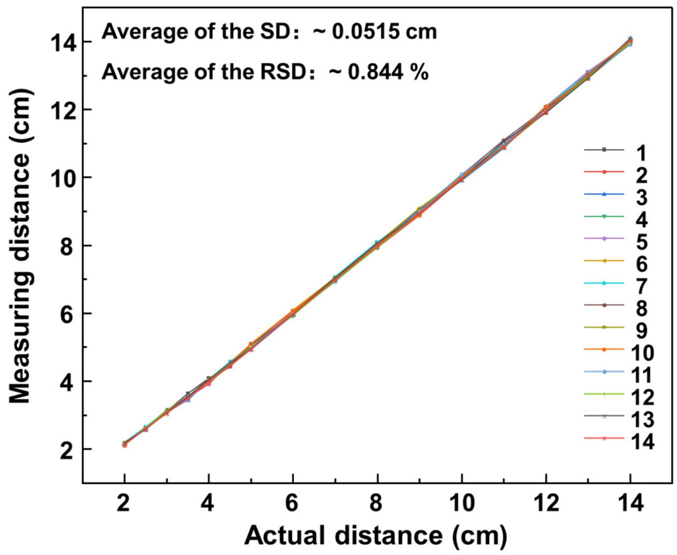

Fig. S2 Repeatability and stability testing of the ultrasonic sensor.

To verify the repeatability and long-term stability of the ultrasonic sensor, a 14-day system test was conducted within the 2-14 cm measurement range. Two sets of measurements were taken daily, and the average value was used, as shown in Fig. S2. The test results showed that the sensor's SD was 0.0515 cm, with an RSD of 0.844%. These results fully confirm that the ultrasonic sensor has excellent distance accuracy, measurement repeatability, and stability during long-term use.

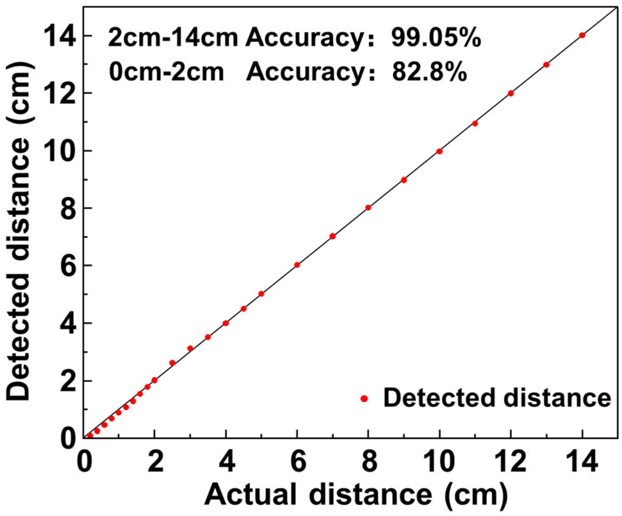

Fig. S3 Comparison between actual distance and detected distance from the ultrasonic sensor.

The ultrasonic sensor based on pMUTs is employed to achieve precise sensing at distances above 2 cm. The measurement accuracy of the ultrasonic sensor is shown in the Fig. S3. The measurement accuracy of the ultrasonic sensor varies significantly across different distance ranges: within the 2-14 cm range, the measurement accuracy reaches 99.05% based on the relative error, while in the 0-2 cm range, the accuracy drops to 82.8%. This difference in accuracy is primarily due to the physical limitations of the ultrasonic sensor at close-range distances, including near-field acoustic effects, time-domain aliasing between transmitted and received signals, and amplified multipath interference. Therefore, a sensor module based on multimodal perception fusion was designed to achieve accurate full-range proximity and contact sensing.

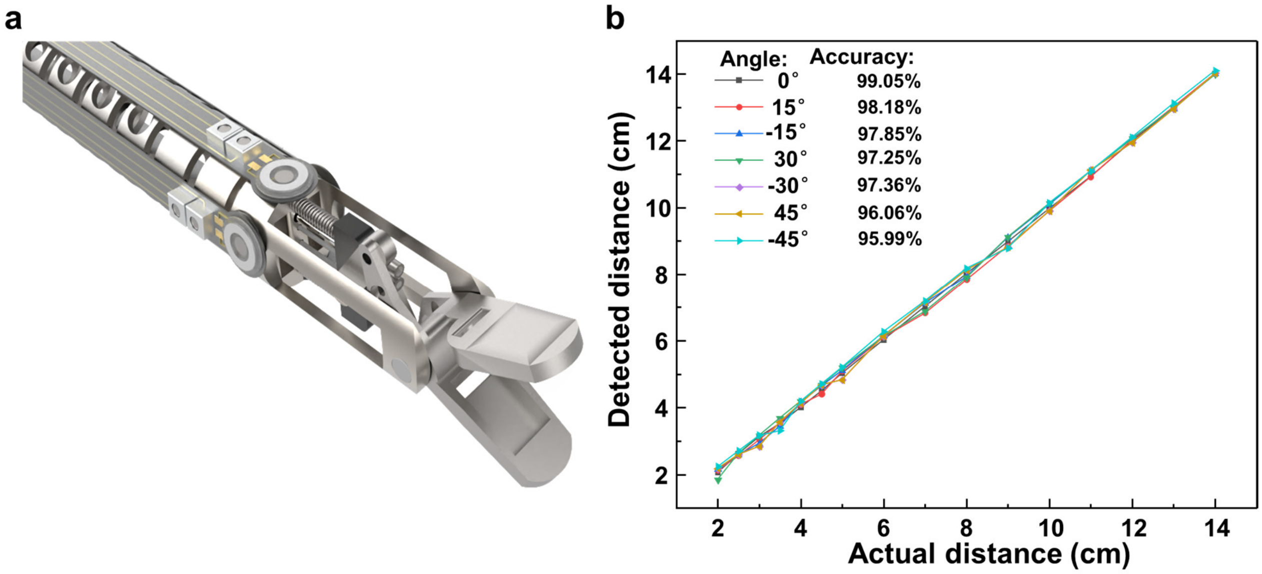

Fig. S4 (a) Surgery robot integrated with four full-range proximity-tactile sensors. (b) Measurement accuracy of the ultrasonic sensor at different measurement angles.

Table S1 Absolute error of the ultrasonic sensor at different angles. (cm)

	45°	30°	15°	0°	-15°	30°	-45°
2cm	0.240	-0.153	0.137	0.057	0.165	0.179	0.176
2.5cm	0.214	0.132	0.064	0.118	0.087	0.099	0.336
3cm	0.178	0.166	0.047	0.127	-0.065	-0.160	0.199
3.5cm	-0.183	0.191	0.061	0.023	-0.055	0.072	-0.141
4cm	0.195	0.188	0.098	0.007	0.164	0.128	0.209
4.5cm	0.215	0.174	-0.088	0.000	0.151	0.183	0.258
5cm	0.214	0.190	0.094	0.023	0.112	-0.168	0.254
6cm	0.279	0.124	0.112	0.023	0.144	0.141	0.165
7cm	0.197	-0.121	-0.169	0.027	0.133	0.157	-0.118
8cm	0.187	-0.104	-0.159	0.020	-0.066	0.129	-0.190
9cm	-0.214	0.118	-0.146	-0.023	0.106	-0.185	0.270
10cm	0.147	0.126	-0.093	-0.024	0.097	-0.097	-0.168
11cm	0.101	0.094	-0.075	-0.063	0.104	0.125	0.130
12cm	0.112	0.061	0.031	-0.003	0.075	-0.056	-0.109
13cm	0.127	-0.019	0.022	-0.007	-0.035	-0.046	0.116
14cm	0.108	-0.013	0.025	0.007	0.011	0.025	0.105

During surgery, human tissues may approach the robot from any angle, which necessitates the investigation of the sensor's performance when approaching tissue at different angles. Four full-range proximity-tactile sensors can be integrated at four directions of the robot, as shown in Fig. S4a. In the case of four sensors, each sensor would be responsible for a 90° angular range of detection. Therefore, it is sufficient to ensure that the sensor achieves accurate measurements within a ±45° angular range.

The performance of the ultrasonic sensor was studied. The sensor was fixed at the center position of

a rotation lift platform, and the glass reflective plate was placed at azimuth angles of ±45°, ±30°, ±15°, and 0°. At each azimuth angle, the distance between the reflective plate and the sensor was gradually increased from 2 cm to 14 cm. Table S1 lists the absolute error between the measurement distance of the sensor and the actual distance at different angles and distances. Fig. S4b shows the comparison between the measured distance and the actual distance at different angles. At various angles, the ultrasonic sensor still has good detection accuracy. At angles within ±45°, the distance measurement accuracy of the ultrasonic sensor exceeds 95%, which meets the required detection accuracy.

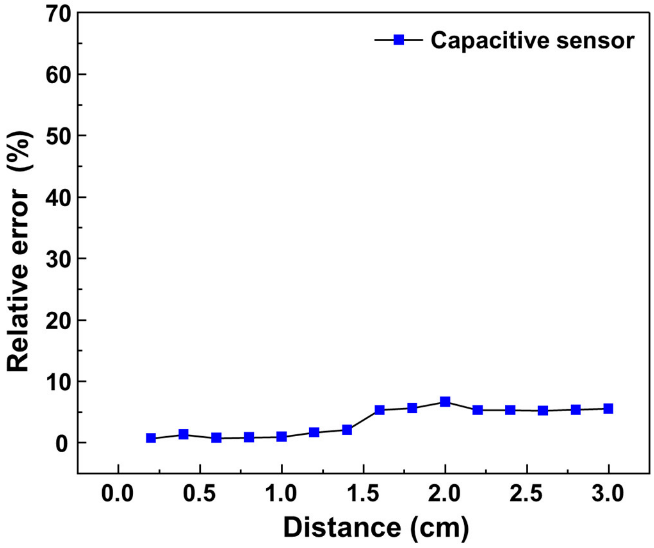

Fig. S5 Relative measurement error of the capacitive sensor at different distances.

The proposed capacitive sensor achieves distance measurement accuracy of 96.47% and relative error of 3.53%. In this work, the average relative error was used to evaluate the accuracy of the capacitive sensor in a measurement range of 0-30 mm. Ten repeated measurements at each distance were conducted, and the sensor detection results were recorded. The detection results were compared with the true values to calculate the relative errors. Ten repeated measurements were conducted at each distance, and the sensor detection results were recorded. The measured values were compared with the true values to calculate the relative errors. The average relative error of the ten measurements was then determined as the relative error at the distance. (Equation S3) The relative error of the sensor is calculated as the average of relative errors across all measured distances, from which the sensor's measurement accuracy can be derived, (Equation S4)

$$R = \frac{1}{n}\sum_{i=1}^{n}\frac{|x - x_i|}{x} \times 100\% \quad \text{(S3)}$$

$$Accuracy = 1 - \frac{1}{k}\sum_{j=1}^{k} R_j \quad \text{(S4)}$$

where n is the number of detections (n=10), x is the actual value, x_i is the detection value, and R is the mean relative error of the specific distance, k is the number of detected distances, R_j is the relative error at j^{th} distance. Detection errors of the sensor at different distances are shown in Fig.S5. The capacitive sensor exhibited a relative error of 3.53%, corresponding to a measurement accuracy of 96.47%.

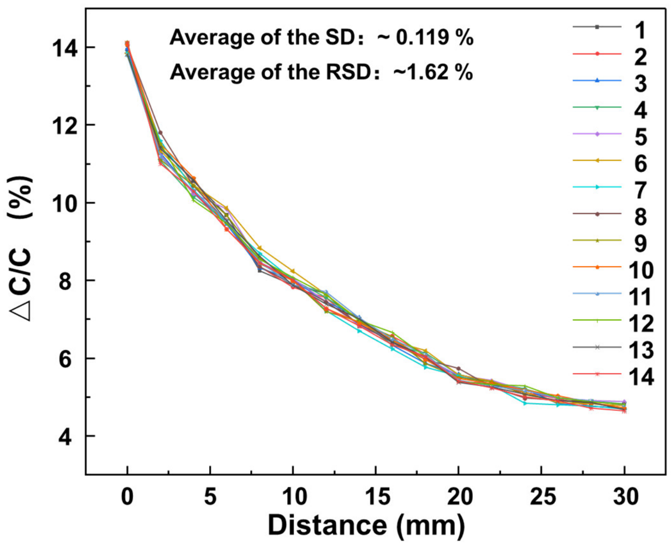

Fig. S6 Repeatability and stability testing of the capacitive sensor.

To evaluate the repeatability and long-term stability of the capacitive sensor, a continuous test was conducted over a 14-day period within the 0-30 mm measurement range. Two independent measurements were taken daily, and the average value was adopted and the results are shown in Fig. S6. The sensor demonstrated excellent stability throughout the entire testing period, with an average standard deviation (SD) of 0.119% and a relative standard deviation (RSD) of 1.62%, fully demonstrating the sensor's reliable repeatability and long-term stability.

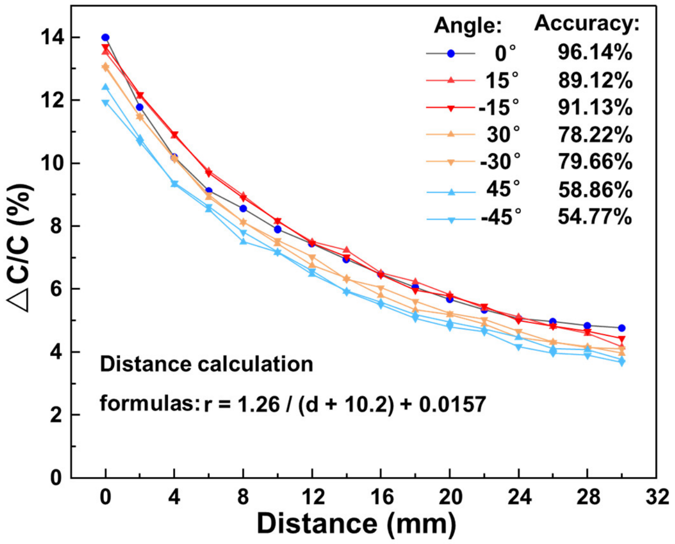

Fig. S7 Measurement accuracy of the capacitive sensor at different measurement angles.

To verify the distance measurement performance of the capacitive sensor at different angles, we also conducted experimental tests on the relative change of capacitance as the hand approached the sensor, with a measurement range of 0-30 mm (testing angles included ±45°, ±30°, ±15°, and 0°), as shown in Fig. S7. The experimental results show that within the ±15° range, the capacitive sensor's measurement accuracy is relatively high. At larger angle ranges the measurement accuracy decreases. Human tissues which approach to the sensor are often a whole-body cavity wall or human organs, whose size is very large relative to the sensor. When its distance from the sensor is less than 1.5 cm, its relative position with the sensor is a large angle range rather than a certain angle. There must be a part within the ±15° range facing the sensor, Therefore, the measurement accuracy of the sensor can be guaranteed.

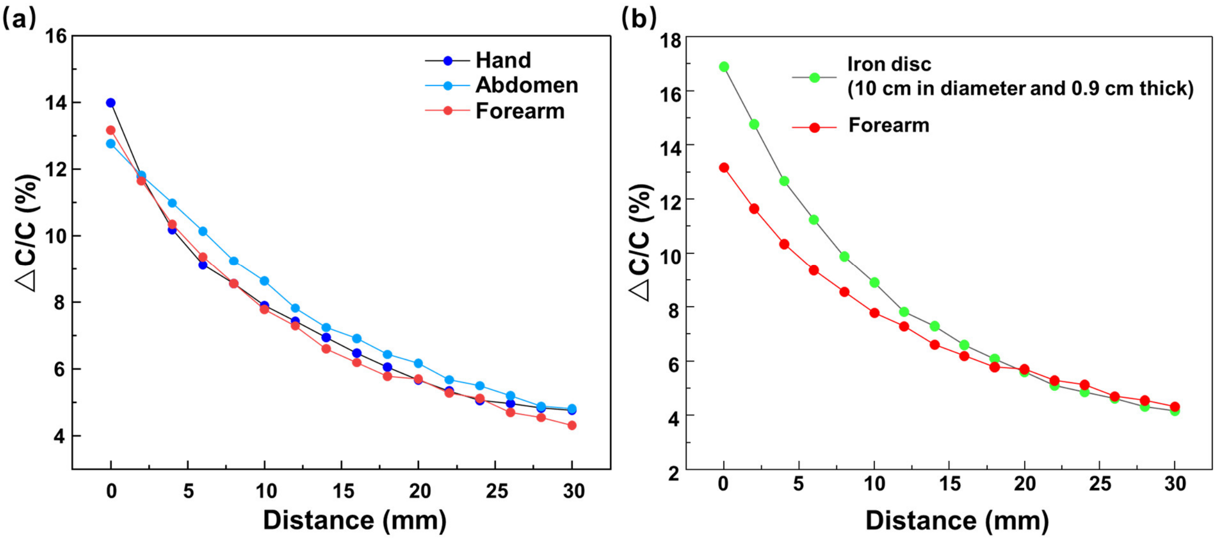

Fig. S8 (a) Capacitive change when different human tissues approach the capacitive sensor. (b) Relationship curve of capacitance change with forearm and iron disc proximity for the capacitive sensor.

The electrical properties of an object, particularly its dielectric constant, and its geometric shape indeed have an impact on the response of the capacitive sensor. When an object with a large dielectric constant approaches the sensor, it results in a more significant change in capacitance, thereby enhancing the sensor's sensitivity. Furthermore, the geometric size of the object will affect its ability to change the electric field distribution around the sensor, and the larger size of objects forming a stronger coupling interaction with the sensor’s electrodes, thereby increasing the electric field modification.

To investigate the impact of different electrical properties of human tissues on the sensitivity of the capacitive sensor, we conducted tests with the hand, abdomen, and forearm. Fig. S8a shows the response of the sensor to the three tissues is close, which is due to the small difference in the overall dielectric constant of the different human tissues. The sensor demonstrated slightly higher response to hand and forearm than that to abdominal area. This difference primarily arises from the tissue compositions: hand and forearm regions contain a higher proportion of muscle tissue with higher dielectric constant, whereas the abdominal area contains a higher proportion of fat tissue with lower dielectric constant. In order to further explore the influence of electrical characteristics of objects on sensor response, we performed the same experiments on the iron disc and the forearm, and the results are shown in Fig. S8b. The results reveal the response of the sensor to the iron disc is greater than that to the forearm, because of the difference in the dielectric constant.

To verify the impact of geometric size on the measurements of the capacitive sensor, we performed

tests on iron discs with different diameters, as shown in Fig. S9. The results indicate that the iron disc with a diameter of 10 cm caused a larger capacitive change compared to that with a diameter of 3 cm. The increase in object geometric size enhances the response of the capacitive sensor. Larger objects can create a stronger electric field coupling with the sensor electrodes.

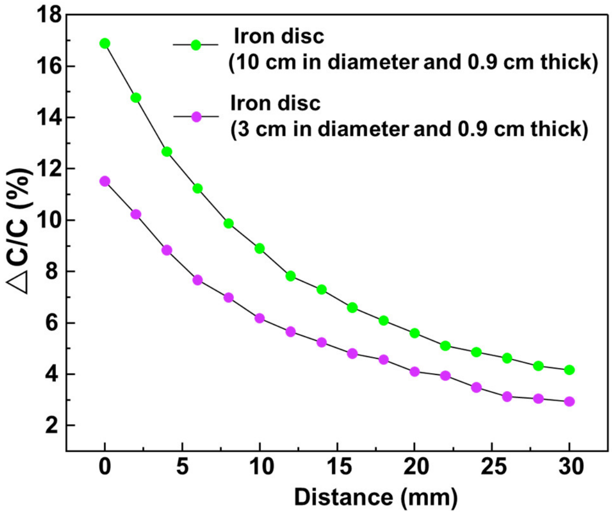

Fig. S9 Capacitive response of the sensor to iron discs with different sizes.

Since the application scenario of the proposed sensor involves RMIS, objects which approach to the sensor are often a whole-body cavity wall or human organs, such as the abdominal wall, whose size is very large relative to the sensor. In this case, the shape differences between different tissues can be neglected. In addition, the difference of electrical properties of different human tissues has little impact on the sensor response, as shown in Fig. S8a. Therefore, the proposed sensor has good detection accuracy in RMIS.

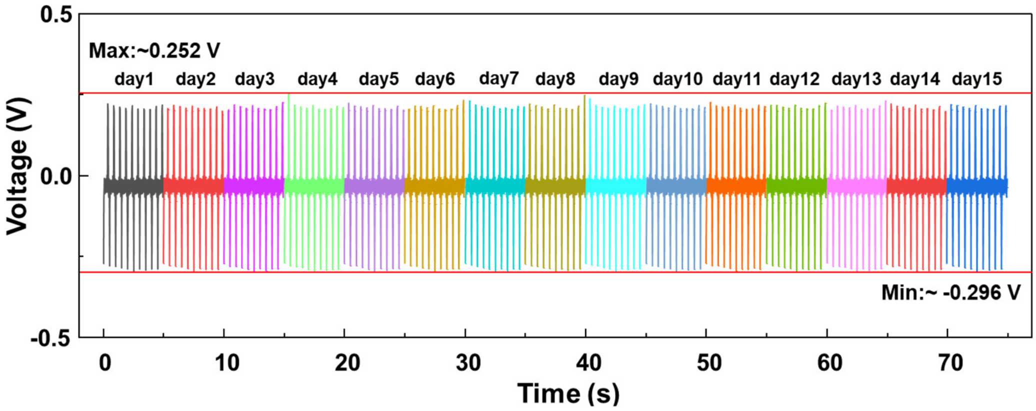

Fig. S10 Repeatability and stability testing of the triboelectric sensor.

For the repeatability and stability testing of the triboelectric sensor, a pressure of 0.5 N was applied to the sensor, and its output voltage signal was monitored in real-time. The test lasted for 15 days. The measurement results as shown in Fig. S10 indicate that the amplitude of the triboelectric signal was highly consistent, with almost no significant variation in the waveform. The signal amplitude ranged from -0.296V to 0.252V, demonstrating extremely high consistency and stability. These results indicate that the triboelectric sensor is capable of providing stable signal output under repeated loading conditions, with good repeatability and long-term stability.

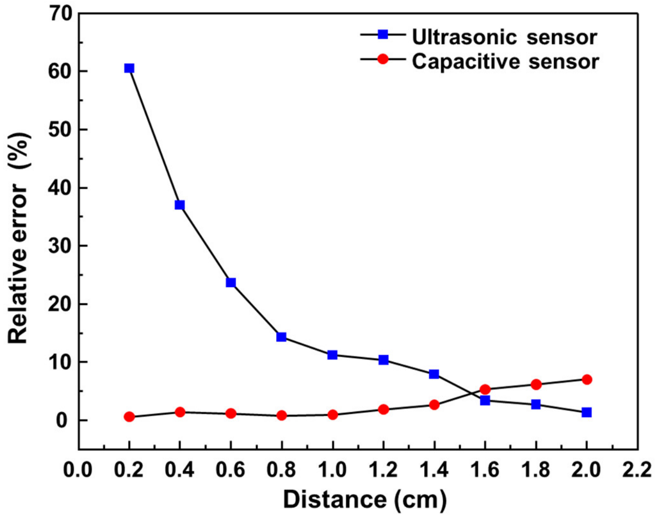

Fig. S11 Comparison between measurement errors of capacitive sensor and ultrasonic sensor.

A comparative analysis was conducted between ultrasonic and capacitive sensors to evaluate their detection accuracy. Fig. S11 shows the comparison between the relative errors of the capacitive and ultrasonic sensors in the range of 0.2 cm to 2 cm. For distances within 1.55 cm, the capacitive sensor's measurement error is smaller than that of the ultrasonic sensor, while for distances larger than 1.55 cm, the ultrasonic sensor has a smaller detection error. The main reason for this difference is that the capacitive sensor working principle relies on changes in the electric field, making it highly sensitive in the near-field range. On the other hand, the ultrasonic sensor achieves more accurate measurements over long-range distances through the emission and reception of sound waves. Therefore, the ultrasonic sensor is used for long-range proximity above 1.5 cm, the capacitive sensor is used for close-range proximity within 1.5 cm, and the triboelectric sensor is responsible for tactile detection.

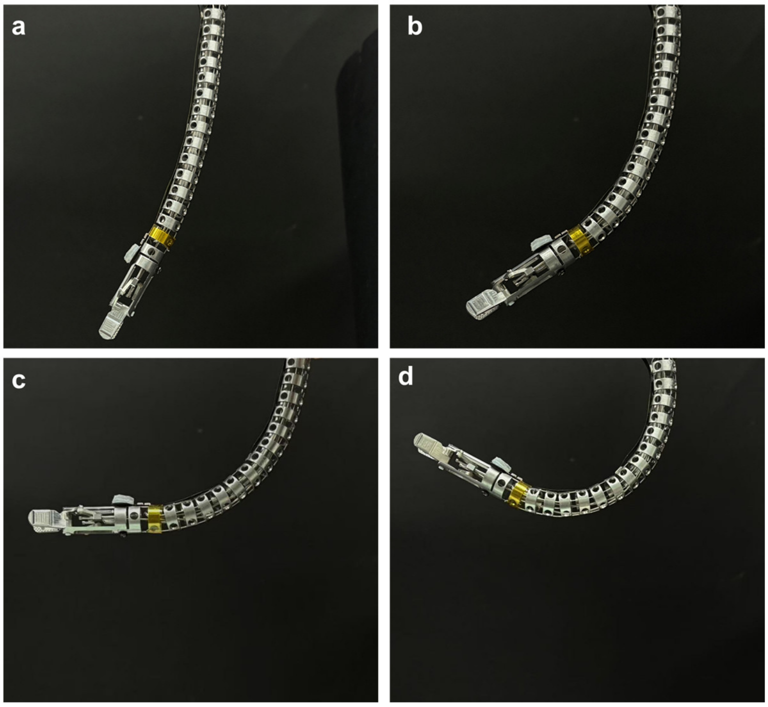

Fig. S12 Continuum surgical robot at different bending angles.

The sensor is fixed onto a flexible printed circuit board, which is then adhered to the surface of the robot's end effector. This design not only allows for good compatibility with the shape of the continuum robot but also minimizes the impact on the robot's flexibility. Fig. S12 shows the performance of the continuum robot integrated with the sensor at different bending angles. It can be seen that the designed sensor is highly compatible with the shape of the continuum robot, and its impact on the robot's movement is negligible.

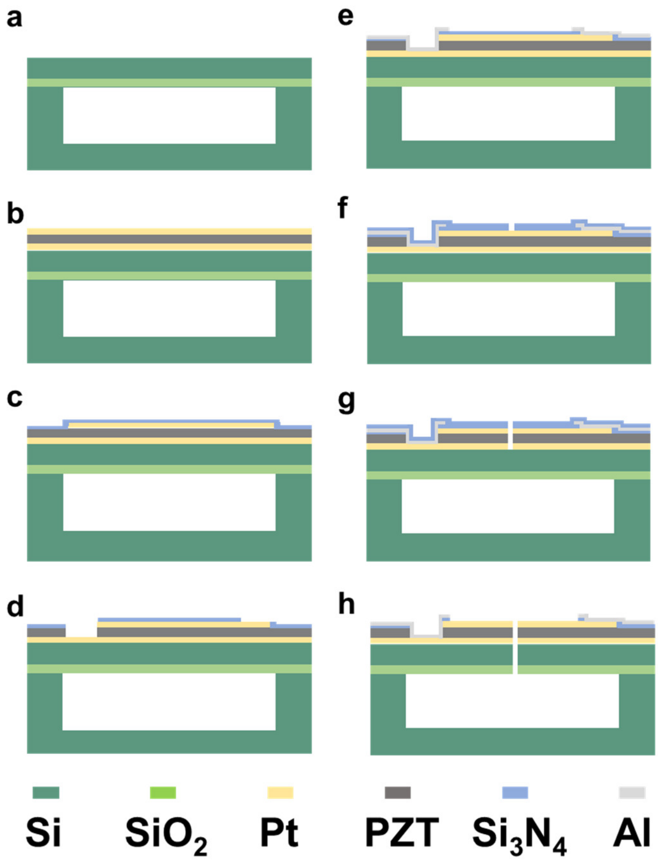

Fig. S13 Fabrication process of the pMUT.

The pMUT is fabricated based on a cavity-SOI (Silicon-On-Insulator, SOI). The specific process flow is as follows: Fabrication starts with a cavity-SOI wafer, which is formed by bonding an SOI wafer with a 1 μm SiO_2 insulating layer, a 5 μm Si structural layer, and a Si substrate with a 20 μm cavity in a vacuum. The cavity corresponds to the pMUT diaphragm position (Fig. S13a). A 0.4 μm Pt bottom electrode, a 2 μm PZT piezoelectric layer, and a 0.4 μm Pt top electrode are deposited via magnetron sputtering (Fig. S13b). The top electrode is patterned through dry etching, followed by the deposition of a Si_3N_4 mask (Fig. S13c). Next, the Si_3N_4 mask and PZT piezoelectric layer are etched to open the electrical connection pathway to the Pt electrodes (Fig. S13d). Subsequently, a 0.2 μm thick Al layer is deposited and etched via magnetron sputtering to form the leads for the pMUT top and bottom electrodes (Fig. S13e). The following describes the process for creating etched hole on the diaphragm. Si_3N_4 is deposited again and patterned (Fig. S13f). The Pt top electrode, PZT and Pt bottom electrode are etched in sequence (Fig. S13g). Then, the Si and SiO_2 insulating layers are then etched to create the etched hole, and the surface Si_3N_4 layer is removed (Fig. S13h). The etched hole connects the internal cavity of the chip to the atmosphere, enabling the diaphragm to vibrate under acoustic wave excitation.

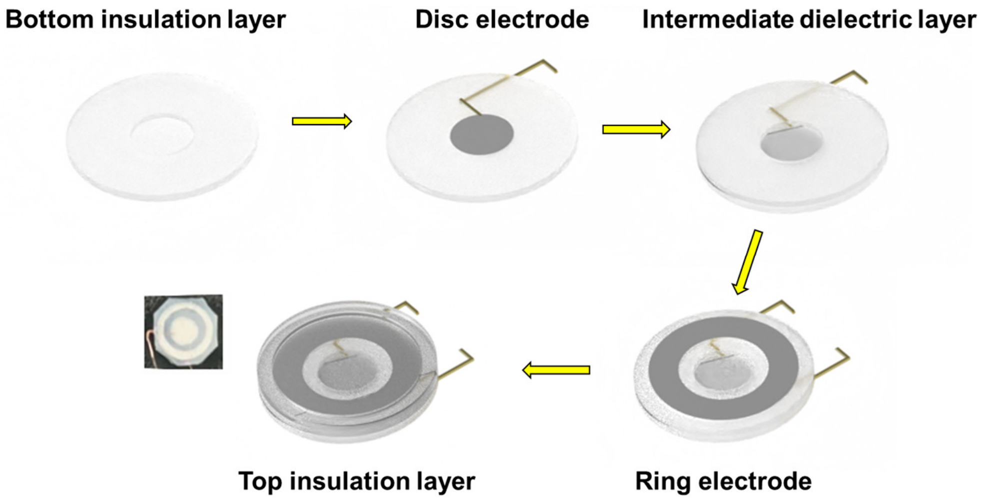

Fig. S14 Fabrication process of the integrated capacitive and triboelectric sensor.

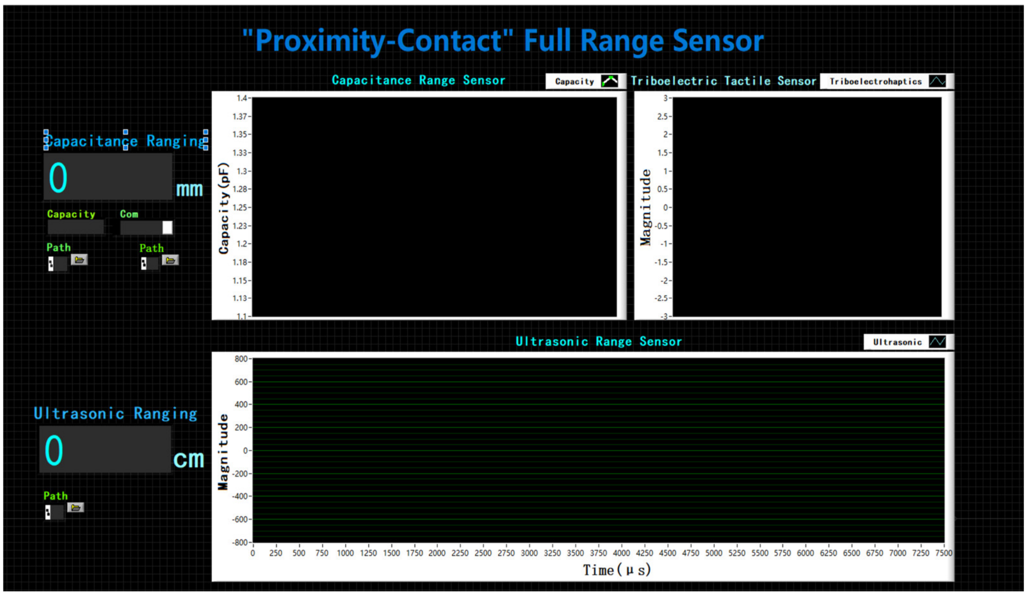

Fig. S15. The LabVIEW visualization interface.

Fig. S15 shows the LabVIEW visualization interface for three types of sensor signals. Through this interface, real-time and accurate reading, processing, and display of the three types of sensor data are achieved, implementing a long-distance, short-distance, and tactile perception system.

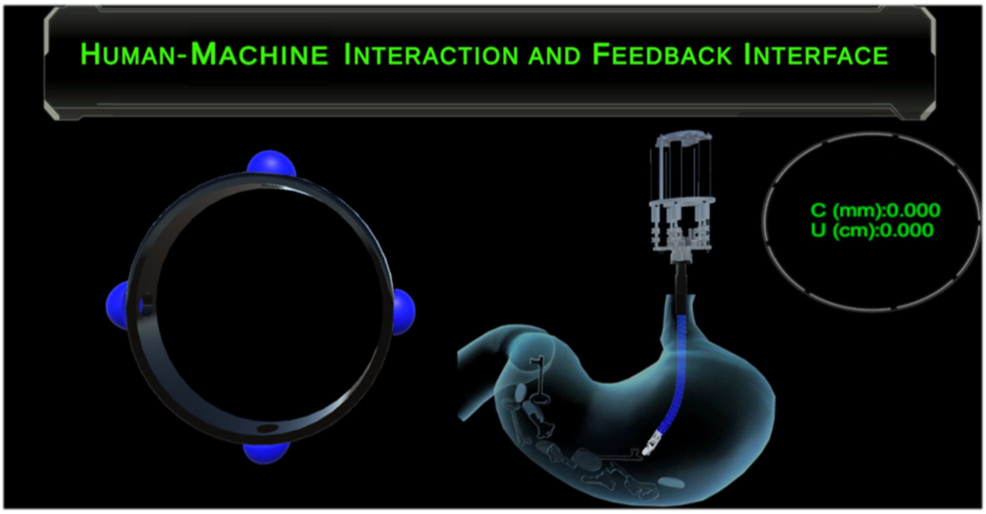

Fig. S16. Unity digital twin interface.

Fig. S16 shows the Unity digital twin interface we designed, which includes models corresponding to the actual force-feedback wristband and the continuum robot. Communication is established through the Transmission Control Protocol (TCP), allowing the interface to retrieve sensor data and robot pose from the continuum robot control system, with the model controlled via scripts. The wristband model responds to vibrations from the actual wristband by triggering corresponding position flashes and sound alerts, providing the operator with intuitive directional vibration feedback. Additionally, as the operator controls the continuum robot, the robot's pose changes. Since the operator may not directly perceive the robot's shape, synchronizing the pose of the continuum robot model with the actual robot enables safer operation. Furthermore, the interface also displays real-time data from ultrasonic long-range distance sensors and capacitive short-range distance sensors, offering the operator continuous distance feedback.

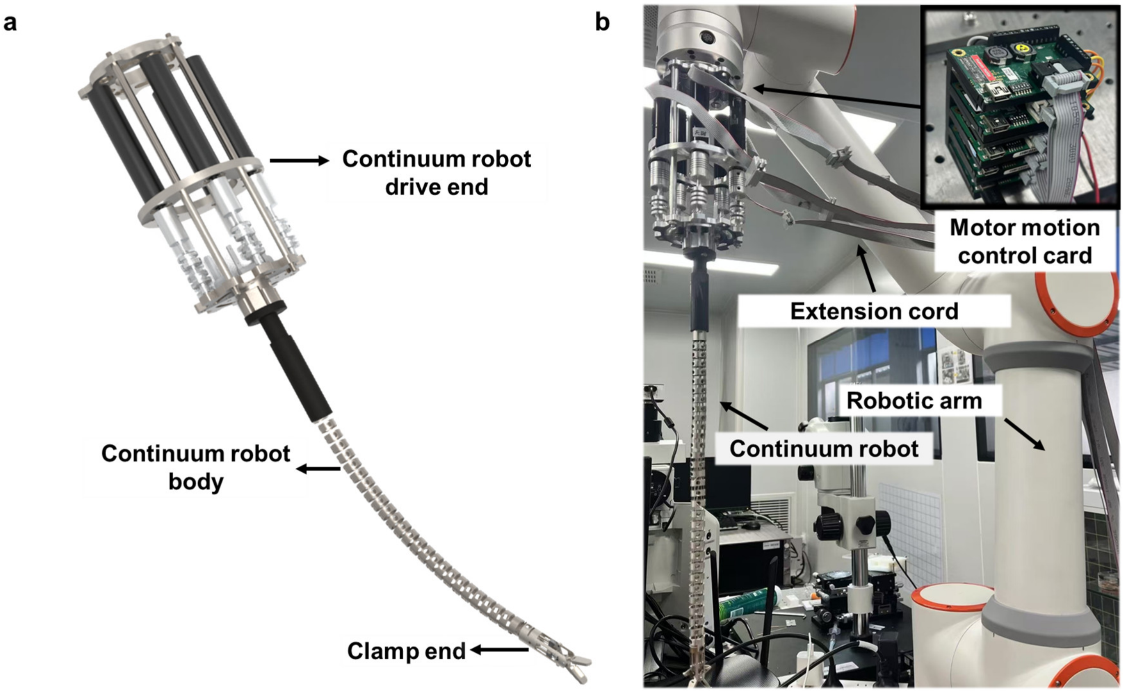

Fig. S17 (a)The detailed design of continuum surgical robot; (b) The physical image of the continuum robot integrated at the end effector of the robotic arm.

The overall design of the continuum robot is shown in Fig. S17a, which includes the continuum robot drive end, the continuum robot body, and the gripper end. Fig. S17b displays the physical image of the continuum robotic arm integrated at the end effector of the robotic arm.

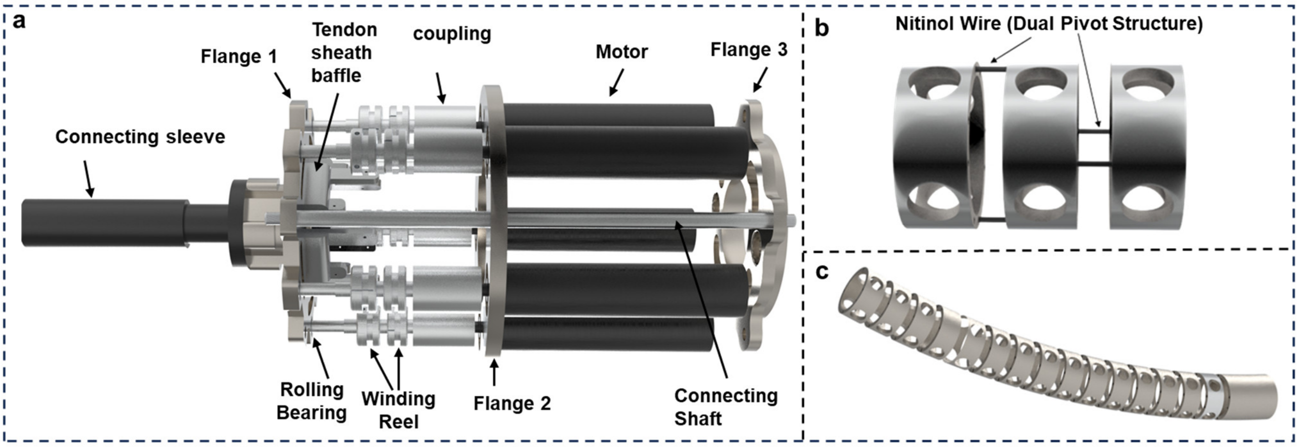

Fig. S18 (a) Continuum robot drive end; (b) Dual-pivot joint bending unit for continuum robot; (c) Bending joint segment of continuum robot.

The drive end of the continuum robot mainly consists of components such as motors, couplers, winding reels, and rolling bearings (Fig. S18a). The drive mechanism works by the motor rotating to drive the winding reel, which in turn moves the wire to enable the bending and gripping functions of the continuum robot. The robot's body uses a dual-pivot joint structure, where adjacent discs are connected by Nitinol wire to form the basic bending unit (Fig. S18b). Multiple bending units are linked in series through dual-pivot joints to form a bending joint segment of the robot's body, achieving two degrees of freedom in bending (Fig. S18c). The robot body designed in this study consists of two bending joint segments.
